# Quantitative measurements of esophageal varices using computed tomography for prediction of severe varices and the risk of bleeding: a preliminary study

**DOI:** 10.1186/s13244-022-01189-5

**Published:** 2022-03-14

**Authors:** Shang Wan, Yuhao He, Xin Zhang, Yi Wei, Bin Song

**Affiliations:** 1 Department of Radiology, West China Hospital, Sichuan University, No.37, Guoxue Alley, Chengdu, 610041 People’s Republic of China; 2 Department of Neurosurgery, Third People’s Hospital of Chengdu, Chengdu, 610031 People’s Republic of China; 3 Pharmaceutical Diagnostic Team, GE Healthcare, Life Sciences, Beijing, 100176 People’s Republic of China

**Keywords:** Computed tomography, Quantitative parameters, Esophageal varices, Esophageal variceal bleeding, Endoscopy

## Abstract

**Background:**

We aimed to assess whether the quantitative parameters of esophageal varices (EV) based on computed tomography (CT) can noninvasively predict severe EV and the risk of esophageal variceal bleeding (EVB).

**Methods:**

A total of 136 endoscopically confirmed EV patients were included in this retrospective study and were divided into a non-conspicuous (mild-to-moderate EV, *n* = 30) and a conspicuous EV group (severe EV, *n* = 106), a bleeding (*n* = 89) and a non-bleeding group (*n* = 47). EV grade (EVG), EV diameter (EVD), cross-sectional surface area (CSA), EV volume (EVV), spleen volume (SV), splenic vein (SNV), portal vein (PV), diameter of left gastric vein (DLGV), and the opening type of LGV were measured independently using 3D-slicer. Univariate and multivariate logistic analysis were used to determine the independent factors and the receiver operating characteristic (ROC) curves were performed to evaluate the diagnostic performance.

**Results:**

The difference of EVG, EVD, CSA, EVV, DLGV, SNV between the conspicuous and non-conspicuous EV group were statistically significant (*p* < 0.05), area under the curves (AUCs) of them for predicting severe EV were 0.72, 0.772, 0.704, 0.768, 0.707, 0.65, with corresponding sensitivities of 70.3%, 63.5%, 50%, 74.3%, 52.7%, 48.6%, specificities of 71.4%, 85.7%, 100%, 71.4%, 81%, 81%, respectively. EVG, CSA (odds ratio 3.258, 95% CI 1.597–6.647; 1.029, 95% CI 1.008–1.050) were found to be independent predictive factors. However, there was no significant difference of the included indices between the bleeding and non-bleeding group (*p* > 0.05).

**Conclusions:**

CT can be used as a noninvasive method to predict the severity of EV, which may reduce the invasive screening of endoscopy.

## Key points

1. CT-derived quantitative parameters can be used to predict severe esophageal varices.
2. New indices revealed accuracy comparable to that of the previous ones.
3. CT may serve as a reliable method to supplement endoscopy.

## Background

As the most relevant complication of portal hypertension in patients with liver cirrhosis, esophageal varices (EV) are present in nearly 50% of cirrhotic patients [1, 2]. Additionally, gastrointestinal hemorrhage from dilated varices is a leading cause of death in cirrhotic patients, with a mortality rate as high as 30% [3]. Therefore, predicting the severity of EV and identifying the risk of esophageal variceal bleeding (EVB) are critical to minimize complications and improve overall survival in cirrhotic patients.

Current guidelines recommend using endoscopy to identify the severity of EV and the risk of EVB. Additionally, some treatments, such as band ligation and endoscopic variceal ligation (EVL), can be performed. However, frequent screening of the condition will also significantly aggravate the risk of iatrogenic bleeding, along with poor patients’ compliance and additional financial burden [1, 4]. Thus, a noninvasive tool for evaluating EV is urgently needed in clinical management.

Computed tomography (CT) is now recognized as a noninvasive evaluation tool for EV, which can provide a comprehensive and visualized assessment for the varix and collateral vessels, with the advantage of better compliance and reduction of pain and the risk of gastrointestinal bleeding associated with endoscopy. Studies have found that measuring the liver lobe volume using magnetic resonance imaging can be used to identify the presence or absence of EV [5, 6]; however, the specific grade of EV and the risk of bleeding that are clinically relevant have not been fully investigated. Recent studies have found that some markers of the varix can help diagnose EV [7–9], such as EV diameter, which was the most widely discussed, and other parameters of the varix, such as EV volume throughout the lower esophagus, cross-sectional surface area of EV, and the degree of EV, have not been discussed. In addition to dilated varices, other collateral vessels, such as the splenic vein, can be established to compress the elevated portal pressure resulting from the increase in intrahepatic resistance, and clinical manifestations, such as splenomegaly and ascites, consequently will be presented [10]. These vessels and complications may have implications on the varices. Meanwhile, comprehensive evaluation of EV integrating varix-relevant indices, collateral vessels and clinical manifestations with noninvasive CT measurements remains lacking, and some relevant parameters that might be effective have not been fully investigated.

In this study, we assessed whether CT-derived quantitative parameters can noninvasively predict the severity of varices and risk of bleeding. Furthermore, we attempted to determine new parameters that could help evaluate EV and determine whether CT can be used as a supplementary monitoring tool to endoscopy.

## Methods

### Patient population

This study was approved by the Institutional Review Board, and written informed consent was waived. This study was conducted following the Declaration of Helsinki. In this retrospective study, we searched the medical records in our hospital to identify consecutive patients with EV who underwent contrast-enhanced CT within 4 weeks of endoscopy from December 2017 to January 2020. The exclusion criteria were as follows: (a) patients with prior variceal treatment (e.g., nonselective β-blockers, band ligation, and EVL) before admission, (b) patients with imaging results that revealed portal vein emboli, (c) patients with histopathological outcomes that were confirmed as hepatocellular carcinoma, and (d) patients with a history of splenectomy, hepatectomy or portal-azygous disconnection. In this study, 136 patients with EV were included and divided into a non-conspicuous (mild-to-moderate EV) and a conspicuous EV group (severe EV) according to the endoscopic results, a bleeding and a non-bleeding group according to the bleeding history. As well, all patients were divided into the training and validation groups at a ratio of 7:3 using a random sampling method; the validation cohort was used to assess the stability of the result [11, 12].

### CT image acquisition

The individuals under study underwent contrast-enhanced CT using one of the following systems: Sensation 64 CT (Siemens, Munich, Germany) or Sensation 16 CT (Siemens, Munich, Germany). Triple-phase CT examinations were conducted, including non-enhanced, arterial, and portal vein phases. The abdomen scouts were acquired from the dome of the diaphragm to the iliac crests. The arterial phase of the same region started at approximately 20–30 s after the administration of contrast agent, followed by the portal phase (30–40 s). Reconstructions were performed on a GE Advantage Windows 3D workstation (GE Healthcare, Waukesha, WI, USA) with the reconstitution thickness set at 1–2 mm. The detailed scanning parameters were listed as follows: tube voltage, 120 or 100 kVp; tube current, 150–600 mA; slice thickness, 1.25 mm; and pitch, 1.375. All patients received an intravenous, nonionic contrast agent (iodine concentration, 370 mg/mL; volume, 1.5–2.0 mL/kg of body weight; contrast type, iopromide injection (Bayer Pharma AG, Leverkusen, Germany)) at a rate of 3–5 mL/s. Then, 20-mL saline was injected after contrast injection.

### CT image analysis

3D Slicer (version 4.10.1; Boston, USA), which is equipped with accurate 2D and 3D measurement tools, was used for the measurement. The included indices were EV grade (EVG), EV diameter (EVD), cross-sectional surface area (CSA), EV volume (EVV), spleen volume (SV), splenic vein (SNV), portal vein (PV), diameter of the left gastric vein (DLGV), and the opening type of LGV; these indices were measured independently by two abdominal radiologists (X.J.L. and W.W.Z.) with more than 10 years’ experiences in analyzing CT images, the final results are the consensus negotiated by the two radiologists, with a third reviewer (C.W.Y.) adjudicating on disagreements. Both observers were blinded to patient physical findings, laboratory data, earlier imaging findings and endoscopic results.

**Fig. 1:**
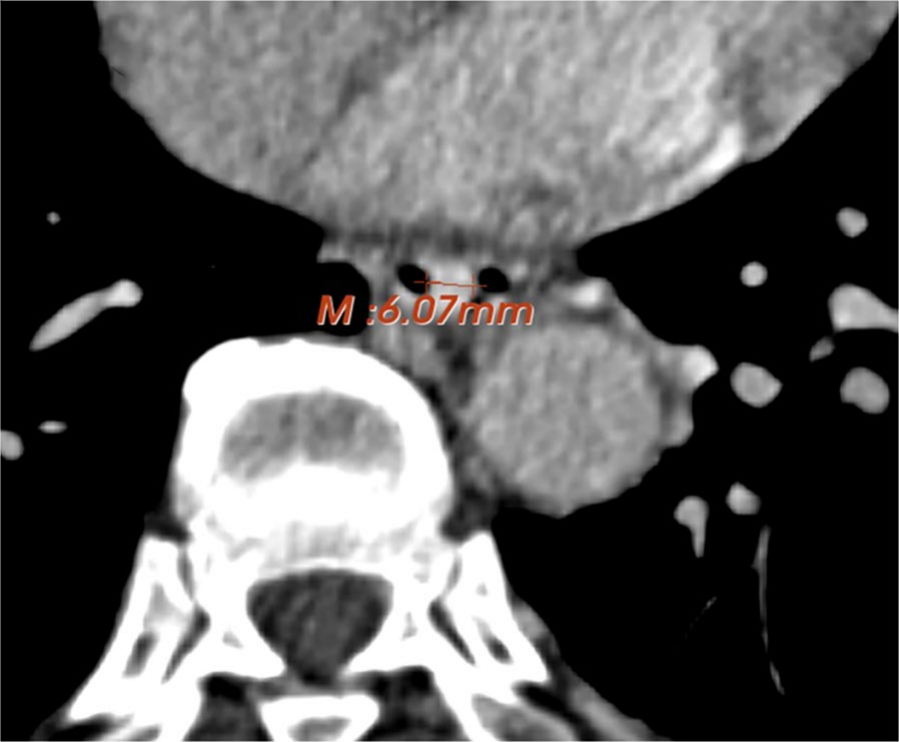
Severe EV according to endoscopy in a 66-year-old man with liver cirrhosis (severe of endoscopy, maximum minor axis 6.07 mm)

The portal venous phase was chosen as the observation period. In measuring EVD, CSA, and EVV, the observation range was 5 cm above the hiatus of the lower esophagus because most dilated veins in cirrhotic patients are located at this site [13]. An image was selected at a point where the varices appeared largest, and the observers measured the short-axis diameter and the area surface of the largest visible esophageal varix [8]. Figure 1 shows the measurement of EVD. The measurement of EVV indicated the volume of the most conspicuous varix within the observation range.

The EV grading system on CT images was according to the criteria proposed by Kim [3], classifying EVs as I–IV mainly according to the EVD and distribution of varices around the inner wall. The DLGV was measured 1 cm from the beginning of the portal or splenic vein opening, SNV is measured at the midpoint, and SV is measured using an automatic software [10]. The opening type of LGV was classified as opening from the portal vein, opening from the splenic vein and opening from the junction of the portal and splenic veins [14].

### Endoscopic study

A Fujinon EG 485 (Fujinon, Saitama, Japan) or OlymPus CV240 (Olympus Optical Co. Ltd., Tokyo, Japan) electronic endoscope was used for EV detection and grading. The grade of EV was divided as follows according to the form (F) and absence or presence of the red color sign (RC): mild (F1,RC–), moderate (F1,RC+ or F2,RC–), and severe (F2,RC+, F3,RC+ or F3,RC–), which were recorded following the General Rules for Study of Portal Hypertension (The Japan Society for Portal Hypertension, second Edition, 2004) [15] and endoscopic diagnosis and treatment standard trial plan for esophagogastric varices (Chinese Society of Digestive Endoscopy, 2000) [16].

### Statistical analysis

Statistical analyses were performed using R (version 3.5.1). In this study, continuous variables were compared using the Mann–Whitney *U* test or Student’s *t*-test according to data distribution, and categorical variables were compared using the chi-square test. Univariate and multivariate logistic regression analyses were performed to screen the independent risk factors. Univariate analyses were performed first, and only parameters found to have statistical significance were used for further stepwise multivariate logistic regression.

The sensitivity and specificity of all included indices in the conspicuous study and EVB study were calculated using receiver operating characteristic (ROC) curve analysis. The area under the ROC curve (AUC) indicates the diagnostic efficiency of these indices for predicting EV severity and risk. *P* values of less than 0.05 were used to indicate statistically significant differences.

## Results

According to the inclusion and exclusion criteria, 21 patients were excluded from the study, and eight patients had a history of hepatic carcinoma, eight patients had a history of EVL and five patients had a history of splenectomy. Finally, 136 patients with EV (including 86 males and 50 females) were evaluated in this study, of whom 95 comprised the training cohort and 41 comprised the validation cohort. According to the Child–Pugh classification, the whole cohort consisted of 38 class A, 63 class B and 35 class C patients. The aetiology of portal hypertension was post-hepatic cirrhosis in 82 patients, alcoholic cirrhosis in 34 patients, primary biliary cirrhosis in 13 patients, mixed cirrhosis in five patients, autoimmune hepatic cirrhosis in one patient, and cryptogenic cirrhosis in one patient. The patients’ characteristics are shown in Table 1. No significant differences in the clinical characteristics were observed between the datasets.

**Table 1:** Demographics of patients among different datasets

Characteristic	Training cohort (*n* = 95)		Validation cohort (*n* = 41)		Estimate risk*	*p* value
	Non-conspicuous (*n* = 21)	Conspicuous (*n* = 74)	Non-conspicuous (*n* = 9)	Conspicuous (*n* = 32)		
Age, mean ± SD, years	56.3 ± 14.7	60.4 ± 12.4	54.7 ± 14	52.5 ± 12.5		
Gender, *n* (%)						
Male	9 (42.8)	45 (60.8)	6 (66.7)	26 (81.2)	1	0.144
Female	12 (57.2)	29 (39.2)	3 (33.3)	6 (18.8)	0.48 (0.18,1.29)	
Etiology, *n* (%)						
Post-hepatic cirrhosis	12 (57.1)	4 (44.4)	48 (64.9)	18 (56.2)	/	0.998
Alcoholic cirrhosis	6 (28.6)	4 (44.4)	14 (18.9)	10 (31.2)	/	
Combined cirrhosis	1 (4.8)	0 (0)	2 (2.8)	2 (6.3)	/	
Primary biliary cirrhosis	2 (9.5)	1 (11.2)	8 (10.8)	2 (6.3)	/	
Autoimmune hepatic cirrhosis	0 (0)	0 (0)	1 (1.3)	0 (0)		
Cryptogenic cirrhosis	0 (0)	0 (0)	1 (1.3)	0 (0)		
Child–Pugh class, *n* (%)						
Class A	8 (38.1)	3 (33.3)	18 (24.3)	9 (28.1)	1	0.195
Class B	8 (38.1)	2 (22.2)	38 (51.4)	15 (46.9)	2.11 (0.68–6.53)	
Class C	5 (23.8)	4 (44.5)	18 (24.3)	8 (25)	1.6 (0.44–5.84)	

*n*: number of patients, age values were expressed as mean ± standard deviation, *: the estimated risk of patients’ characteristics with univariable analysis

### Endoscopic findings

By analyzing the findings obtained by endoscopy, which was considered the reference standard in this study, we found that of the 136 patients, 18 (13.2%) had mild EV and 12 (8.8%) had moderate EV (these patients were considered the non-conspicuous EV group (*n* = 30)). Moreover, 106 (78%) patients had severe EV, who were considered the conspicuous EV group (*n* = 106). The grading system and endoscopic results of this study are presented in Table 2.

**Table 2:** The grading system for esophageal varices and endoscopic results of our study

Grade	Endoscopic criterion		*n*
	Form (F)	Red color sign (RC)	
Mild	F1	RC−	18
Moderate	F1	RC+	12
	F2	RC−	
Severe	F2	RC+	106
	F3	RC+ or RC−	

*n*, number of patients; F, form; RC, red color sign

### Quantitative parameters for evaluation of EV severity

**Fig. 2:**
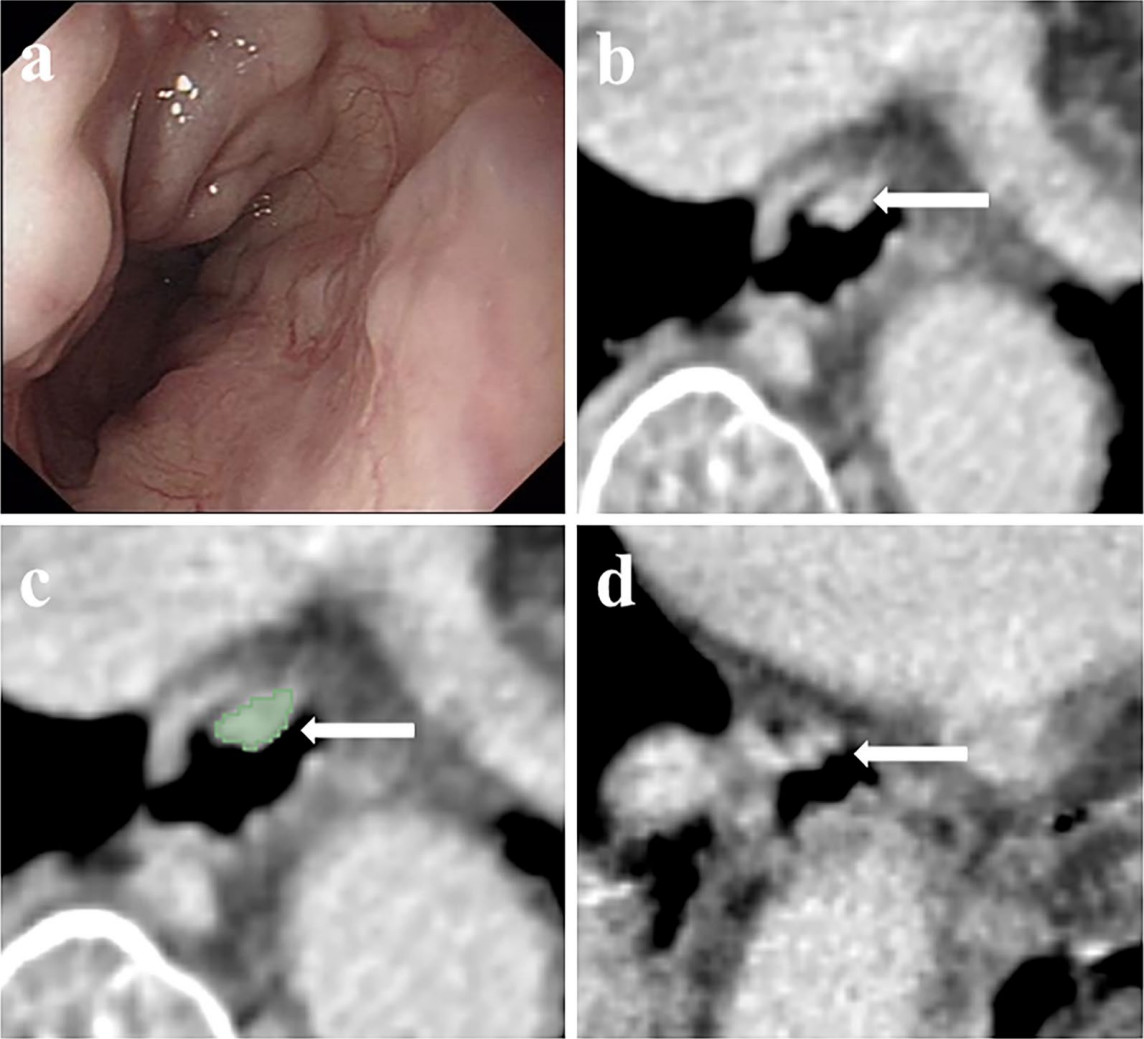
Moderate EV according to endoscopy in a 74-year-old woman with liver cirrhosis (moderate of endoscopy, maximum minor axis 4.23 mm). **a** The endoscopic image showed tortuous dilation of varices protruding from the esophageal wall (arrow); **b** The cross-sectional surface area (CSA) of EV in the transverse section is depicted in 3D slicer; **c** CSA of EV can be clearly displayed by using 3D slicer measurement tool (dyeing area, arrow); **d** 3D reconstruction image shows the dilated varices in the lower esophageal (arrow)

**Fig. 3:**
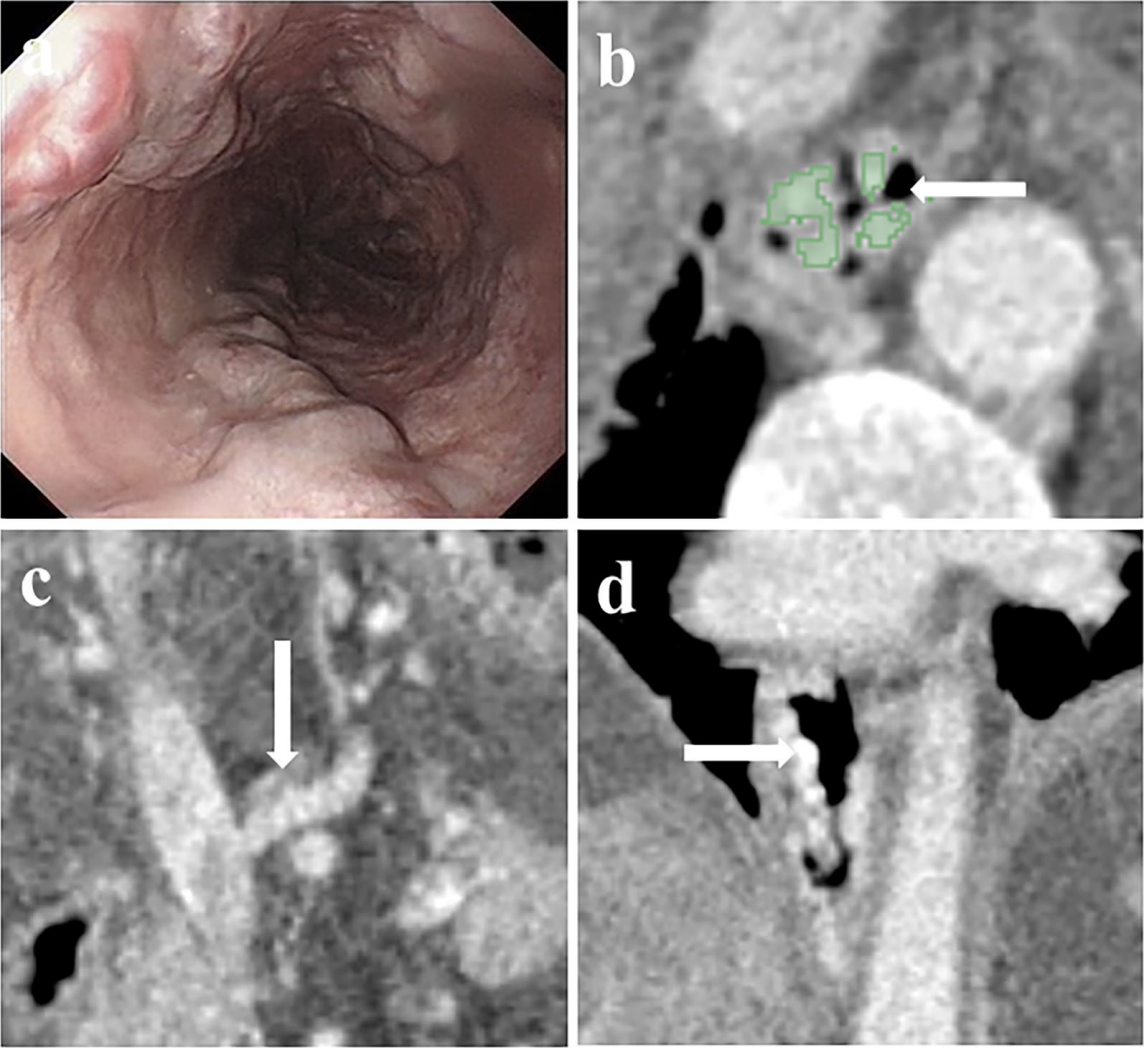
Severe EV according to endoscopy in a 68-year-old man with liver cirrhosis (severe of endoscopy, maximum minor axis 6.28 mm). **a** The endoscopic image shows severe nodular varices; **b** the cross-sectional surface area (CSA) of EV (dyeing area) in the transverse section is depicted in 3D slicer; **c** the axial CT image shows the left gastric vein (LGV) originating from the portal vein (arrow); **d** 3D reconstruction has a satisfactory performance in visualizing severe EV of the lower esophagus (arrow). EV, esophageal varices

On CT images, varices were clearly visible in the lower esophagus. The target region was characterised by local circumferential esophageal wall thickening with nodular enhancements protruding into the luminal space, intraluminal protrusions or irregularities and nodular enhancements within the wall presenting as distinct enhancing nodular or linear lesions abutting the surface or protruding into the luminal space. Paraesophageal and submucosal varices were also clearly displayed on CT images. Patients with moderate EV (Fig. 2) and severe EV (Fig. 3) were clearly depicted.

In the evaluation of the severity of EV, univariate analysis showed the difference in all indices, except for the SV and PV, between the two groups were statistically significant (*p* < 0.05) (Table 3). EVD, CSA, EVV, SNV, and DLGV of the conspicuous group were higher than those of the non-conspicuous group (6.71 ± 2.76 mm vs. 4.44 ± 1.39 mm, 92.98 ± 87.64 mm^2^ vs. 28.44 ± 19.35 mm^2^, 3410.69 ± 3319.72 mm^3^ vs. 951.43 ± 807.38 mm^3^, 11.53 ± 3.21 mm vs. 9.95 ± 1.86 mm, 6.31 ± 2.22 mm vs. 4.90 ± 2.53 mm, respectively). Moreover, the difference in the distribution of EVG between the two groups was statistically significant (*p* = 0.001). However, the differences in the PV and SV between the two groups were not statistically significant (15.70 ± 3.13 mm vs. 14.86 ± 1.88 mm and 8.4 × 10^5^ ± 4.7 × 10^5^ mm^3^ vs. 6.6 × 10^5^ ± 3.1 × 10^5^ mm^3^_,_ respectively). Furthermore, no statistically significant difference in the opening types of LGV was found between the two groups (*p* > 0.05) (Table 4).

**Table 3:** Quantitative parameters on CT in the study of EV severity

Parameters	Non-conspicuous	Conspicuous	Univariate analysis OR (95% CI)	*p* value	Multivariate analysis OR (95% CI)	*p* value
EVD (mm)	4.44 ± 1.39	6.71 ± 2.76	1.702 (1.231, 2.355)	0.001	1.28 (0.8, 2.06)	0.302
CSA (mm^2^)	28.44 ± 19.35	92.98 ± 87.64	1.030 (1.010, 1.050)	0.003	1.029 (1.008, 1.050)	0.006
EVV (mm^3^)	951.43 ± 807.38	3.4 × 10^3^ ± 3.3 × 10^3^	1.001 (1.000, 1.001)	0.006	1.0003 (0.9993, 1.0012)	0.609
MPV (mm)	14.86 ± 1.88	15.70 ± 3.13	1.117 (0.928, 1.345)	0.242		
SNV (mm)	9.95 ± 1.86	11.53 ± 3.21	1.214 (1.009, 1.462)	0.040	1.14 (0.89, 1.44)	0.279
DLGV (mm)	4.90 ± 2.53	6.31 ± 2.22	1.389 (1.057, 1.826)	0.018	1.18 (0.87, 1.59)	0.256
SV (mm^3^)	6.6 × 10^5^ ± 3.1 × 10^5^	8.4 × 10^5^ ± 4.7 × 10^5^	1.000 (1.000, 1.000)	0.107		
EVG					3.258 (1.597, 6.647)	0.001
EVGI	5 (23.81%)	5 (6.76%)	3.258 (1.597, 6.647)	0.001		
EVGII	10 (47.62%)	17 (22.97%)				
EVGIII	6 (28.57%)	52 (70.27%)				

Parameters except EVG are expressed as mean ± standard deviation

OR: odds ratio; CI: confidence interval, EV: esophageal varices, EVG: EV grade, EV diameter: EVD, cross-sectional surface area: CSA, EV volume: EVV, spleen volume: SV, diameter of left gastric vein: DLGV

**Table 4:** The opening types of LGV in the study of EV severity

	Opening type of LGV		
	Portal vein	Splenic vein	Junction of portal-spleen vein
Non-conspicuous EV, *n* (%)	10 (47.62%)	10 (47.62%)	1 (4.76%)
Conspicuous EV, *n* (%)	45 (60.81%)	24 (32.43%)	5 (6.76%)
OR (95%CI)	1.875 (0.685, 5.132)		2.083 (0.215, 20.171)
*p* value	0.221		0.526

EV: esophageal varices, OR: odds ratio, CI: confidence interval

Multivariate logistic analysis showed that EVG and CSA (odds ratio (OR) 3.258; 95% confidence interval (CI) 1.597–6.647; OR 1.029; 95% CI 1.008–1.050) (*p* < 0.05) were independent predictive factors of severe EV. Other indices were found to have no statistical significance in the multivariate regression model (Table 3).

**Fig. 4:**
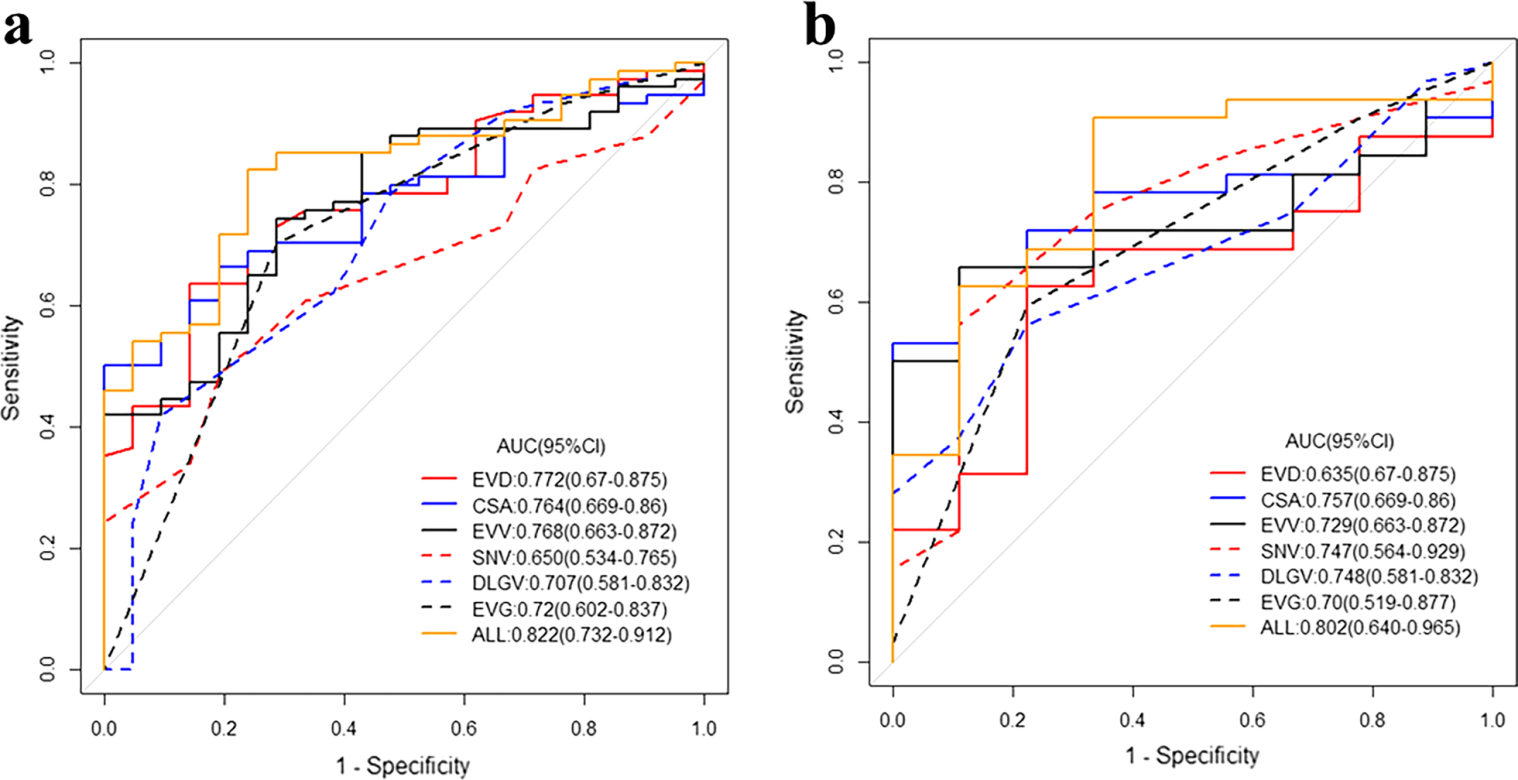
Receiver operating characteristic (ROC) analysis of each index for predicting severe EV in the training (**a**) and validation cohort (**b**), the area under the curves (AUC) and 95% confidence interval (CI) are also displayed

Table 5 shows the diagnostic performance of the indices for predicting severe EV. ROC analysis of each index in both cohorts is depicted in Fig. 4. In the training cohort, the AUCs of EVG, EVD, CSA, EVV, DLGV, and SNV for differentiating mild-to-moderate EV from severe EV were 0.72, 0.772, 0.704, 0.768, 0.707, and 0.65, respectively. The sensitivities of these indices were 70.3%, 63.5%, 50%, 74.3%, 52.7%, and 48.6%, respectively, while the specificities were 71.4%, 85.7%, 100%, 71.4%, 81%, and 81%, respectively.

**Table 5:** Diagnostic performance of variables for identifying severe EV

Variables		Training cohort (*n* = 95)							Validation cohort (*n* = 41)									
		AUC	ACC			SPE	SEN		AUC		ACC		SPE		SEN		Cut-off value	
EVG	0.72			0.705	0.714			0.703		0.698		0.643		0.778		0.594		III (I, II, III)
EVD	0.772			0.684	0.857			0.635		0.635		0.537		0.778		0.469		5.58 (mm)
CSA	0.704			0.611	1			0.5		0.757		0.512		1		0.375		70.39 (mm^2^)
EVV	0.768			0.737	0.714			0.743		0.729		0.659		0.444		0.719		1083.8 (mm^3^)
DLGV	0.707			0.589	0.81			0.527		0.748		0.634		0.889		0.562		7 (mm)
SNV	0.65			0.558	0.81			0.486		0.75		0.634		0.889		0.562		12 (mm)

AUC: area under the curve, ACC: accuracy, SPE: specificity, SEN: sensitivity, CI: confidence interval, the remaining abbreviations are shown in Table 3

### Quantitative parameters for evaluating EVB

The patients were divided into the bleeding (*n* = 89) and non-bleeding (*n* = 47) groups according to the presence or absence of bleeding history. Univariate analysis indicated no significant difference in EVG, EVD, CSA, EVV, SNV, DLGV, PV and SV between the two groups (*p* > 0.05) (Table 6). Moreover, no significant difference in the opening type of LGV was found between the two groups (Table 7).

**Table 6:** Quantitative parameters on CT in the study of EVB

Parameters	Non-bleeding group	Bleeding group	*p* (student *t* tests)	Univariate analysis OR (95% CI)	*p* value
EVD (mm)	6.21 ± 2.54	6.21 ± 2.77	0.991	1.001 (0.849, 1.180)	0.991
CSA (mm^2^)	67.45 ± 66.98	83.41 ± 87.92	0.391	1.003 (0.997, 1.009)	0.391
EVV (mm^3^)	2.2 × 10^3^ ± 2.2 × 10^3^	3.1 × 10^3^ ± 3.4 × 10^3^	0.206	1.000 (1.000, 1.000)	0.211
MPV (mm)	15.64 ± 3.32	15.46 ± 2.75	0.785	0.979 (0.842, 1.138)	0.782
SNV (mm)	10.64 ± 3.30	11.40 ± 2.91	0.267	1.090 (0.936, 1.270)	0.266
DLGV (mm)	5.89 ± 2.33	6.04 ± 2.38	0.776	1.029 (0.850, 1.245)	0.773
SV (mm^3^)	7.6 × 10^5^ ± 4.4 × 10^5^	8.2 × 10^5^ ± 4.5 × 10^5^	0.595	1.000 (1.000, 1.000)	0.591
EVGI	2 (7.14%)	8 (11.94%)	0.899	0.909 (0.470, 1.759)	0.777
EVGII	9 (32.14%)	18 (26.87%)			
EVGIII	17 (60.71%)	41 (61.19%)			

Parameters except EVG are expressed as mean ± standard deviation and abbreviations are shown in Table 3

**Table 7:** The opening types of LGV in the study of EVB

	Opening type of LGV		
	Portal vein	Splenic vein	Junction of portal-spleen vein
Non-bleeding group, *n* (%)	18 (64.29%)	7 (25.00%)	3 (10.71%)
Bleeding group, *n* (%)	37 (55.22%)	27 (40.30%)	3 (4.48%)
OR (95% CI)	0.533 (0.195, 1.455)		0.259 (0.043, 1.574)
*p* value	0.219		0.142

OR: odds ratio, CI: confidence interval

## Discussion

Esophageal varices are the most clinical relevant complication of cirrhosis that develop from the elevated pressure in the portal venous system [1]. Despite of the limitation of the invasive nature and expensive cost, endoscopy still remains the reference standard for the detection and diagnosis of esophageal varices, and varices identified at endoscopy are in only the superficial portions of intrinsic veins, ignoring the remaining venous plexuses of esophagus [17]. Whereas, endoscopic ultrasonography (US) can demonstrate not only superficial veins but also the perforating and extrinsic veins that cannot be identified at endoscopic examination, all those veins constitute the esophageal venous systems [18, 19]. Similarly to endoscopic US, CT can demonstrate all the venous plexuses [20], it allows direct and comprehensive visualization of the distal esophagus, additionally, CT is less invasive in comparison with the interventional procedure of the endoscopic US.

In the past few decades, noninvasive imaging approaches have shown the potential for the diagnosis of chronic liver disease [1, 21], of which, contrast-enhanced ultrasound (CEUS) plays an important role in differentiating cysts from solid focal liver lesions that can be a challenge for traditional imaging modalities [22], as well, it shows accurate depiction of microvasculature and microvasculature of the target organ because of its specifically blood pool nature [23], the previous literature also suggested that it may be used to assess the portal venous system and can predict the presence of EV [24]. Likewise, trans-abdominal ultrasound and Doppler have shown promising results for the diagnosis of portal hypertension as regular and non-radiated screening tools [25, 26], and another study suggested trans-abdominal US may have a role to play in the detection of EV in patients with portal hypertension [17], which firstly indicated the manifestation and the diagnostic criteria of EV based on trans-abdominal US and encouraged further noninvasive assessment of EV.

Recent literatures indicated the important role of CT in the evaluation of portal hypertension and the esophageal varices. The results of this study also suggested that quantitative parameters based on CT may be used to predict the severity of EV, we found that EVG, EVD, CSA, EVV, DLGV, and SNV could be used to predict severe EV in patients with liver cirrhosis, among which EVG, CSA, EVV, and SNV could be used as effective new indices, which have not been previously investigated, and their diagnostic abilities were comparable to those of EVD and DLGV. The prediction model analysis suggested that EVG and CSA were risk factors for severe EV. However, the included indices showed no statistical significance in the EVB study, which may not be able to predict the risk of EVB.

As the most widely investigated index, EVD has been validated to be able to assess EV. Kim et al. have concluded that EVD of 3 mm or larger on CT, as a criterion, could help identify high-risk patients requiring prophylactic therapy [7]. In the training cohort, we found that a threshold of 5.58 mm or larger yielded a sensitivity of 63.5% and a specificity of 85.7% for predicting severe EV, which showed good diagnostic performance (AUC = 0.774); the results of the validation cohort further confirmed the stability of our results, and regular follow-up should be considered in cases with EVD of ≥ 5.2 mm. Our findings were in accordance with ‘A guide to EV treatment for portal hypertension in the USA’ which recommends prophylactic treatment, including EVL and beta-blockers, if the diameter is greater than 5 mm [27].

Presently, no relevant studies have investigated EV severity by evaluating EVG on CT. The results of this study suggested that in the conspicuous EV study, the distribution of EVG showed significant differences between the two groups (*p* = 0.001); particularly the distribution difference in EVG III showed good diagnostic performance (AUC = 0.72, training cohort). EVG seemed more comprehensive than EVD and may provide more information because the classification of which is mainly based on EVD and its distribution on the inner wall of the esophagus [3]. Furthermore, our results demonstrated that a CSA of 70.39 mm^2^ or larger can identify severe EV, which, on CT, yielded a sensitivity and specificity of 50% and 100%, respectively, for the training cohort and 37.5% and 100%, respectively, for the validation cohort. The low sensitivity in both cohorts may be attributed to the structural characteristics of the esophagus; CSA may not accurately reflect the true cross-sectional area in cases where the vein folds or is embedded in the esophageal wall. The multivariate model showed that CSA and EVG were independent risk factors for severe EV (OR, 3.258 and 1.029, respectively), indicating that with the increase in EVG and CSA, that is, from I to III of EVG, and from a small area to a large area of CSA, the incidence of severe EV will increase accordingly.

Most studies have assessed EVD, PV, and DLGV [14, 28, 29]. In this study, as a newly investigated index, EVV demonstrated efficacy comparable to those of EVD and CSA for detecting severe EV. A threshold of 1083.8 mm^3^ was effective for predicting severe EV (yielding 71.4% sensitivity and 74.3% specificity), which might be a useful parameter to predict severe EV on CT. Several advantages of this index were presented in this study. In contrast, volume measurement obviates the need to choose images with the largest visible esophageal varix in the distal esophagus as required for measuring EVD and CSA; as a result, the consistency between observers was high, and the subjectivity of measurement was less than that in other indices. However, volume may provide more information on various veins throughout the lower esophagus, whereas EVD and CSA only reflect the condition of a specific vein, which may be especially valuable if visualization is limited, as in cases where the esophageal wall is collapsed.

This study also demonstrated that DLGV could predict severe EV (AUC = 0.707). A threshold of 7 mm can yield a sensitivity of 52.7% and a specificity of 81%. The relatively high specificity may be attributed to the anatomical relationship between the LGV and PV. Because the LGV is the main branch of the portal vein and is the inflow vein of the lower esophageal vein, with the increase in the pressure of the portal vein, the blood flow into the liver appears to be a reverse flow, leading to accumulated dilation of the portal vein and the dilation of the LGV [10]. As another major branch of the portal vein, the SNV is rarely studied; currently, only one study has shown that the SNV is correlated with EV degree (*p* < 0.05) [30]. This study showed that the SNV could be used as an index to predict EV severity, and this result still needs further verification.

We found no significant difference in the PV between the conspicuous and non-conspicuous groups. As the most common manifestation of portal hypertension, the diameter of the PV increases gradually as the disease progresses, and a collateral circulation can be established to reduce the portal pressure, such as the opening of the umbilical vein and accessory umbilical vein and the dilation of the splenic vein. The degree of the shunt may affect the severity of varices. Therefore, the diameter of the PV may not directly reflect the severity of EV, which should be further investigated more comprehensively.

This study showed no significant difference between the bleeding and non-bleeding groups. Several factors may have caused the results. First, although the consecutive patients in this study were strictly included according to the inclusion and exclusion criteria, patients with severe EV comprised the majority of our population due to the high prevalence of severe EV of our hospital, bringing a potential bias of our study cohort, and large-scale samples with more patients with mild or moderate EV are warranted to obtain high-quality evidence for further validation. Second, the episode of acute bleeding is associated with a series of complex pathophysiological changes; thus, only evaluating quantitative indices on CT could not comprehensively assess the risk of EVB. Further studies integrating CT-derived indices and clinical data should be conducted to explore EVB episodes.

This study has several limitations that should be noted. First, a significant degree of inter- and interobserver disagreement exists due to the subjective nature of endoscopic grading systems [31], and we recommend that further re-evaluation studies should be conducted by trained endoscopists, which may eliminate any potential bias among observers. Additionally, given the retrospective nature of the study, some interval progression or regression of the disease cannot be managed. In the future, a controlled prospective study is needed to provide more accurate information. Finally, quantitative parameters of this study were found not valuable for predicting the risk of variceal bleeding, which is the most clinical relevant event, and a consecutive study of our team has revealed the potential bleeding risk using liver volumetric parameters based on CT [32], however, more further studies are needed to validate the results.

## Conclusion

In conclusion, we found that CT can be used as a reliable method for evaluating the severity of EV and may supplement endoscopy to a certain extent. Furthermore, we found that EVG, CSA, EVV, and SNV can be used as effective parameters to evaluate patients with severe EV, with accuracy comparable to that of EVD and DLGV, which have been previously investigated. Additionally, EVG and CSA were risk predictors of severe EV. Further studies are needed to verify the findings of this study.

## Data Availability

The data are available for scrutiny from external requests.

## Abbreviations

AUC: The area under the ROC curve
CEUS: Contrast-enhanced ultrasound
CI: Confidence interval
CSA: Cross-sectional surface area
CT: Computed tomography
DLGV: Diameter of left gastric vein
EV: Esophageal varices
EVB: Esophageal variceal bleeding
EVD: EV diameter
EVG: EV grade
EVL: Endoscopic variceal ligation
EVV: EV volume
F: Form
ICC: Intra-class correlation coefficient
NSBB: Nonselective β-blockers
OR: Odds ratio
ROC: Receiver operating characteristic
SV: Spleen volume
SNV: Splenic vein
RC: Red color sign
US: Ultrasonography

## References

[1] Garcia-Tsao G, Abraldes JG, Berzigotti A, Bosch J (2017). Portal Hypertensive Bleeding in Cirrhosis: Risk Stratification, Diagnosis, and Management: 2016 Practice Guidance by the American Association for the Study of Liver Diseases. Hepatology.

[2] Groszmann RJ, Garcia-Tsao G, Bosch J (2005). Beta-blockers to prevent gastroesophageal varices in patients with cirrhosis. N Engl J Med.

[3] Kim YJ, Raman SS, Yu NC (2007). Esophageal varices in cirrhotic patients: evaluation with liver Ct. AJR Am J Roentgenol.

[4] Vasudevan AE, Goh KL, Bulgiba AM (2002). Impairment of psychomotor responses after conscious sedation in cirrhotic patients undergoing therapeutic upper gi endoscopy. Am J Gastroenterol.

[5] Li H, Chen TW, Li ZL (2015). Albumin and magnetic resonance imaging-liver volume to identify hepatitis B-related cirrhosis and esophageal varices. World J Gastroenterol.

[6] Yang LB, Xu JY, Tantai XX (2020). Non-invasive prediction model for high-risk esophageal varices in the Chinese Population. World J Gastroenterol.

[7] Kim H, Choi D, Gwak GY (2009). Evaluation of esophageal varices on liver computed tomography: receiver operating characteristic analyses of the performance of radiologists and endoscopists. J Gastroenterol Hepatol.

[8] Miller LS, Banson F, Bazir K (2003). Risk of esophageal variceal bleeding based on endosscopic ultrasound evaluation of the sum of esophageal variceal cross sectional surface area (Csa). Am J Gastroenterol.

[9] Wan S, Wei Y, Yu H (2020). Computed tomographic portography with esophageal variceal measurements in the evaluation of esophageal variceal severity and assessment of esophageal variceal volume efficacy. Acad Radiol.

[10] Zhou H, Chen T, Zhang X (2012). The diameter of the originating vein determines esophageal and gastric fundic varices in portal hypertension secondary to posthepatitic cirrhosis. Clinics (Sao Paulo).

[11] Tan Y, Zhang ST, Wei JW (2019). A radiomics nomogram may improve the prediction of idh genotype for astrocytoma before surgery. Eur Radiol.

[12] Yin P, Mao N, Liu X (2020). Can clinical radiomics nomogram based on 3d multiparametric mri features and clinical characteristics estimate early recurrence of pelvic chondrosarcoma?. J Magn Reson Imaging.

[13] Zhang X, Sun H, Xu J (2013). CT findings of esophageal varices in patients with cirrhosis and portal hypertension predict the risk of first upper gastrointestinal bleeding. Chin J Clin (Electron Edn).

[14] Xin He, Zhongkui H, long Liling, (2012). Clinical application of multi-slice spiral CT portal vein imaging in liver cirrhosis with esophageal varices. J Pract Radiol.

[15] Kawano Y, Sasaki A, Kai S (2008). Short- and long-term outcomes after hepatic resection for hepatocellular carcinoma with concomitant esophageal varices in patients with cirrhosis. Ann Surg Oncol.

[16] Dahong D (2000). Trial scheme of endoscopic diagnosis and treatment of esophageal and gastric varices. Chin J Digest Endoscopy.

[17] Kishimoto R, Chen M, Ogawa H, Wakabayashi MN, Kogutt MS (1998). esophageal varices: evaluation with transabdominal us. Radiology.

[18] Liu JB, Miller LS, Feld RI, Barbarevech CA, Needleman L, Goldberg BB (1993). Gastric and esophageal varices: 20-Mhz transnasal endoluminal us. Radiology.

[19] Caletti G, Brocchi E, Baraldini M, Ferrari A, Gibilaro M, Barbara L (1990). Assessment of portal hypertension by endoscopic ultrasonography. Gastrointest Endosc.

[20] Pham JT, Kalantari J, Ji C, Chang JH (2020). Quantitative Ct predictors of portal venous intervention in uncontrolled variceal bleeding. AJR Am J Roentgenol.

[21] Qi X, Berzigotti A, Cardenas A, Sarin SK (2018). Emerging non-invasive approaches for diagnosis and monitoring of portal hypertension. Lancet Gastroenterol Hepatol.

[22] Corvino A, Catalano O, Corvino F, Sandomenico F, Petrillo A (2017) Diagnostic performance and confidence of contrast-enhanced ultrasound in the differential diagnosis of cystic and cysticlike liver lesions. AJR Am J Roentgenol 209:W119–W127 10.2214/AJR.16.17062 28639831

[23] Qiu L, Zhang X, Liu D, Qian L, Hu X (2016). Contrast-enhanced ultrasonography diagnostic evaluation of esophageal varices in patients with cirrhosis. Ultrasound Q.

[24] Tamano M, Yoneda M, Kojima K (2004). Evaluation of esophageal varices using contrast-enhanced coded harmonic ultrasonography. J Gastroenterol Hepatol.

[25] Maruyama H, Yokosuka O (2017). Ultrasonography for noninvasive assessment of portal hypertension. Gut Liver.

[26] Kim G, Cho YZ, Baik SK, Kim MY, Hong WK, Kwon SO (2015). The accuracy of ultrasonography for the evaluation of portal hypertension in patients with cirrhosis: a systematic review. Korean J Radiol.

[27] Talwalkar JA (2006). Cost-effectiveness of treating esophageal varices. Clin Liver Dis.

[28] Guiqin L, Jing H, Jialin S (2015). value of CT portal vein angiography in predicting esophageal and gastric variceal bleeding in cirrhotic portal hypertension. Chin J Pract Diagn Treat.

[29] Kim H, Choi D, Gwak GY (2010). Evaluation of esophageal varices on liver computed tomography: receiver operating characteristic analyses of the performance of radiologists and endoscopists. J Gastroenterol Hepatol.

[30] Zhiling J, Xuping C, Bing F (2011). Relationship between degree of esophageal varices, diameter of portal splenic vein and child Pugh grade in liver cirrhosis. West China Med.

[31] Paul C (2003). Evaluation of Baveno recommendations for grading esophageal varices. J Hepatol.

[32] Wan S, Wei Y, Zhang X, Yang C, Song B (2021). Ct-derived quantitative liver volumetric parameters for prediction of severe esophageal varices and the risk of first variceal hemorrhage. Eur J Radiol.

